# Adaptive responses of *Gordonia alkanivorans* IEGM 1277 to the action of meloxicam and its efficient biodegradation

**DOI:** 10.3389/fbioe.2025.1603975

**Published:** 2025-07-23

**Authors:** Semyon Tyan, Nadezhda Kostrikina, Vladimir Sorokin, Andrey Mulyukin, Irina Ivshina

**Affiliations:** ^1^ Institute of Ecology and Genetics of Microorganisms, Federal Research Center, Ural Branch of The Russian Academy, Perm, Russia; ^2^ Winogradsky Institute of Microbiology, Research Center of Biotechnology, Russian Academy of Sciences, Moscow, Russia

**Keywords:** non-steroidal anti-inflammatory drugs (NSAIDs), meloxicam, pharmaceutical pollution, *Gordonia*, biodegradation, bacterial cell responses

## Abstract

**Introduction:**

Pharmaceutical contaminants such as meloxicam pose significant environmental risks due to their persistence and toxicity. The biodegradation potential of actinomycetes, particularly representatives of *Gordonia*, offers promising avenues for eco-friendly wastewater treatment. However, the ability of *Gordonia* to fully degrade meloxicam has not been previously demonstrated.

**Methods:**

The biodegradation of meloxicam was investigated using *G. alkanivorans* IEGM 1277 as a model organism. Metabolite identification was performed using liquid chromatography-mass spectrometry (LC-MS). Candidate genes encoding meloxicam-oxidising enzymes were identified via genomic analysis. Adaptive bacterial responses to meloxicam exposure were characterised using atomic force microscopy (AFM), transmission electron microscopy (TEM), and energy-dispersive X-ray spectroscopy (EDX).

**Results:**

*G. alkanivorans* IEGM 1277 successfully decomposed meloxicam into primary metabolites, 5'-hydroxymethyl- and 5'-carboxymeloxicam, which exhibited reduced (eco)toxicity compared to the parent compound. Genomic analysis revealed several candidate genes potentially involved in meloxicam oxidation. Microscopic and spectroscopic analyses demonstrated significant phenotypic and metabolic changes in bacterial cells, indicating adaptive defence mechanisms triggered by meloxicam exposure.

**Discussion:**

This study provides the first evidence of complete meloxicam biodegradation by *Gordonia* and elucidates the underlying enzymatic and adaptive cellular responses. The findings highlight the potential application of *G. alkanivorans* IEGM 1277 in developing efficient and environmentally safe biotechnologies for pharmaceutical wastewater treatment.

## 1 Introduction

The contamination of the environment with pharmaceuticals and its resultant harm constitutes a significant worldwide issue of indisputable relevance (Boxall and Brooks, 2024; Gupta et al., 2024; Wilkinson et al., 2024). Once released, these compounds act as hazardous pollutants, driving extreme conditions and ecotoxic effects (Wang et al., 2024; Meena et al., 2025). Even at environmentally relevant concentrations, residual pharmaceuticals and their transformation products exert acute and chronic adverse impacts on terrestrial and aquatic organisms (Carter et al., 2024; Saeed et al., 2024). The effects of pharmpollutants on many organism groups, particularly microorganisms – primary responders to xenobiotic – remain insufficiently studied, and their adaptive mechanisms are largely unexplored (Świacka et al., 2021). Non-steroidal anti-inflammatory drugs (NSAIDs), such as diclofenac, ibuprofen, ketoprofen, and naproxen, are the most frequently reported and toxic pharmaceutical pollutants (Huynh et al., 2023). Meloxicam (MLX), a widely used NSAID, has been reported to occur in natural ecosystems and living organisms and is thus considered a persistent pharmaceutical pollutant (Zorrilla et al., 2015; Herrero-Villar et al., 2020; Wang et al., 2021). Nevertheless, there are significant knowledge gaps concerning its environmental fate (Wilkinson et al., 2024). As demonstrated by a paucity of studies, the MLX concentrations in different aquatic systems may vary significantly, from nano- to micrograms per 1 L (Gros et al., 2012; Jiménez et al., 2018), although the data on its occurrence in environmental samples for many geographic regions, including Russia, are still absent.

It is important to note that there is a risk of environmental contamination associated with MLX, which can be attributed to its extensive consumption and large-scale production (Ahmed et al., 2005; Bekker et al., 2018). Its main pharmacological action mechanism is associated with the selective blockade of cyclooxygenase-2, which is formed at sites of tissue damage and is responsible for the active synthesis of prostaglandins (Tavares, 2000). Approximately 20 million prescriptions for MLX were issued in the US in 2011, with an annual average of 9 million (Dalal et al., 2017; LiverTox, 2025). MLX is manufactured in India, Germany, the Republic of Belarus, the Russian Federation, and other countries with a broad distribution network across the global market. In Russia, 63.7 million packs of MLX have been sold over the past 25 years (Karateev et al., 2021).

Meloxicam (MLX, CAS# 71125-38-7; C_14_H_13_N_3_O_4_S_2_, 4-hydroxy-2-methyl-N-(5-methyl-2-thiazolyl)-2*H*-1,2-benzothiazine-3-carboxamide 1,1-dioxide, also known as Mobic^®^) is a highly toxic oxicam-class NSAID. The detrimental effects of MLX on invertebrate animals, fish, birds, and mammals have been well-documented. MLX has been demonstrated to cause neuronal damage in the roundworm *Caenorhabditis elegans* (da Silva et al., 2020); to cause oxidative stress in the common carp *Cyprinus carpio* (Sheikhlangi et al., 2023); and to result in DNA damage in mice (da Silva et al., 2022). However, a significant proportion of groups of organisms have not yet been studied in relation to the effects of MLX.

Pharmaceutical compounds persist in aquatic and terrestrial ecosystems due to ineffective removal by conventional physicochemical wastewater treatments (Boxall and Brooks, 2024; Wilkinson et al., 2024). Consequently, alternative remediation strategies are required. Biodegradation, leveraging microbial enzymatic activity for pharmaceutical decomposition, represents the most promising approach (Mishra et al., 2021; Crowther et al., 2024; Shah et al., 2024). This method supports eco-friendly wastewater management and aligns with Sustainable Development Goals by minimising environmental impact (Singh et al., 2024).

It is a conceivable, albeit as yet unexamined, hypothesis that the representatives of *Actinomycetes* have the capacity to destroy MLX. Genus *Rhodococcus* exhibit remarkable metabolic versatility and genomic plasticity, enabling their survival in diverse natural and anthropogenically polluted environments. *Rhodococcus* species, recognised for their extensive capacity to degrade xenobiotics such as drotaverine (Ivshina et al., 2015), diclofenac (Ivshina et al., 2019), and ibuprofen (Ivshina et al., 2021) – pharmaceuticals frequently detected in aquatic ecosystems and identified by the European Commission as posing significant environmental risks (Zhou et al., 2019) – play a critical role in bioremediating contaminated ecosystems. The other related actinomycetes *Gordonia sensu stricto* demonstrates robust adaptability to changing climatic and ecological conditions, with metabolic capabilities that facilitate the degradation of complex organic xenobiotics, including heterocyclic compounds, aromatic hydrocarbons, and polyethylene (Sowani et al., 2017; Sánchez-Suárez et al., 2022; Frantsuzova et al., 2023). It is hypothesised that *Gordonia* multifunctional oxygenase systems would catalyse direct MLX oxidation processes, including the introduction of hydroxyl groups into the molecule’s aromatic ring up to complete decomposition of its chemical structure. However, an issue with the tolerance of different actinobacterial species to MLX remains unresolved.

The objectives of this study were to characterise the response of *Gordonia* strains to MLX, identify the most efficient MLX-degrading strains, and elucidate the underlying degradation mechanisms.

## 2 Materials and methods

### 2.1 Bacterial strains and chemicals

In the study, 20 strains of actinomycetes belonging to different *Gordonia* species were used – *G. alkanivorans* (3 strains), *G. amarae* (1 strain), *G. amicalis* (1 strain), *G. rubripertincta* (14 strains), *G. sputi* (1 strain) from the Regional Specialised Collection of Alkanotrophic Microorganisms (acronym IEGM, the World Federation for Culture Collections # 285, http://www.iegmcol.ru) (Catalogue of Strains, 2025). The selection of bacterial strains was guided by distinctive ecological traits indicative of adaptation to particular environmental niches. We focused on microorganisms from areas affected by human activities to explore how they can help clean up pollution and understand how they cope with stress. This targeted approach not only augments the applied relevance of the study but also contributes to a deeper understanding of the functional diversity and ecological roles of microorganisms within contaminated ecosystems.

Chemical reagents, such as acetonitrile, orthophosphoric acid and *n*-hexadecane, were of chemically and analytically pure grade (Cryochrome, Russia; Merck, Germany; Sigma-Aldrich, United States). A Millipore Simplicity Personal Ultrapure Water System (Millipore, United States) was used to obtain the ultrapure water. MLX was used as a pure pharmaceutical substance (a light yellow powder, poorly soluble in water, odorless, with a purity of 99.99%, BLD Pharmatech Ltd., China).

### 2.2 Cultivation conditions

Bacterial cells were cultured in LB broth (Sigma, United States) with 160 rpm shaking at 28°C for 2 days. After centrifuging the broth cultures for 15 min at 4,500 rpm, they were twice washed in 10 mM phosphate buffer (pH 7.0). Bacterial cells were added to the culture medium to a final concentration of 5 × 10^8^ cells/mL. The concentration of bacterial cells was determined using spectrophotometer Lambda EZ201 (Perkin-Elmer, United States) at a wavelength of 600 nm (OD_600_).

### 2.3 Minimum inhibitory concentrations of MLX

The minimum inhibitory concentrations (MICs) of MLX for actinomycetes was determined using the twofold dilution method in LB broth with 96-well microplates. Initial concentration selection was based on the need to overcome bacterial defences (for instance, decreased cell envelope permeability, active efflux, and enzymatic inactivation of toxicants) and identify potential antimicrobial effects of MLX that were unrelated to anti-inflammatory activity. In this regard, a working dose close to the therapeutic dose (5 g/L) was selected. MLX was introduced at an initial concentration of 5 g/L, followed by a twofold dilution to a final concentration of 0.15625 g/L. 10 μL of bacterial suspension was added to the resulting mixture. Incubation was performed at 28°C for 3 days with agitation on a Titramax 1,000 plate shaker (Heidolph, Germany). At the end of the incubation, the culture medium was stained with 50 µL of a 0.2% iodonitrotetrazolium chloride (INT, Sigma-Aldrich, United States) for a duration of 2 h, thus enabling the INT reduction to insoluble red-violet INT-formazan in the presence of actively respiring cells. The formazan concentration was measured spectrophotometrically at 630 nm sing a microplate reader (Multiskan Ascent, Thermo, Vantaa, Finland) to assess cell viability. OD_630_ serves as an indicator of microbial vitality and bacterial cell resistance (Lan et al., 2019).

### 2.4 Biodegradation of MLX

The mineral medium RS (100 mL in 250-mL Erlenmeyer flasks), used in biodegradation experiments, contained (g/L): K_2_HPO_4_–2.0; KH_2_PO_4_–2.0; KNO_3_–1.0; (NH_4_)_2_SO_4_–2.0; NaCl–1.0; MgSO_4_–0.2; CaCl_2_–0.02, FeCl_3_ × 7H_2_O–0.001. The stock concentrated MLX solution (with 3 × 10^−5^ M NaOH to increase the solubility of MLX) was sterilised by passing through a nylon filter with 0.22 µm pore size (LabFil, China) and added to the RS medium to a final MLX concentration of 0.001% (10 mg/L). The incubation was carried out at a temperature of 28°C with constant stirring 160 rpm. Light-shielded flasks were used to prevent photodegradation and photo-initiated oxidation of MLX. Since MLX cannot serve as the sole carbon and energy source for *Gordonia*, biodegradation was performed with 0.1% (*v/v*) *n*-hexadecane as a co-substrate, providing an accessible carbon and energy source for hydrocarbon-degrading bacteria (Alotaibi et al., 2022). The selected concentration is based on previous data showing that increasing *n*-hexadecane concentration excessively stimulates bacterial growth, whereas decreasing it insufficiently activates metabolic processes (Mishra and Singh, 2012). The controls were: (1) sterile drug solution in RS medium containing *n*-hexadecane (to assess the abiotic MLX degradation); (2) sterile drug solution in RS medium with inactivated *Gordonia* cells (to assess the extent of drug adsorption on bacterial cells), where the bacterial cells were inactivated upon autoclaving at 1.0 atm three times for 20 min; (3) RS medium containing *n*-hexadecane with live cells (to assess its effect on bacterial cells and to distinguish the metabolites resulting from MLX degradation).

In separate experiments, selective inhibition of cytochrome P450-associated monooxygenases was carried out using specific blockers (1-aminobenzotriazole, metyrapone, 4-(methylthio) phenylacetic acid) at concentrations ranging from 0.1 to 1.0 mM to evaluate the impact of CYP450-mediated oxygenase reactions on the degradation process of MLX.

### 2.5 Analytical methods

The quantitative content of MLX in the culture medium was determined using high-performance liquid chromatography (HPLC) on a LC Prominence 20A chromatography column (Shimadzu, Japan) with Discovery^®^ C18 reversed-phase sorbent (25 × 4.6 mm, 5 μm, Supelco, United States) and diode-matrix detector (SPD-M20A). The mobile phase was phosphate buffer (pH 3.5)–acetonitrile in a 60:40 (*v/v*) ratio. The eluent flow rate was 0.5 mL/min, the column temperature was 30°C, and the detection wavelength was 254 nm.

Products of MLX decomposition were identified by liquid chromatography-mass spectrometry (LC-MS) using a LC Prominence instrument (Shimadzu, Japan) and a Luna 3uC18(2) 100A chromatographic column (150 × 3.0 mm) (Phenomenex, United States) with the isocratic elution. The mobile phase was composed of formic acid solution (0.1%)–acetonitrile in an 80:20 (*v/v*) ratio. The eluent flow rate was 0.3 mL/min. Detection in positive ion registration mode was by scanning in the range of *m/z* 50–500. Mass spectrometric detector interface was DUIS. Flow rates of spray gas, heating gas and drying gas were 3, 10 and 10 mL/min, respectively. Temperatures of the interface, desolvation line and heating unit were 200°C, 300°C and 400°C, respectively. Flow rate of drying gas was 10 L/min. Capillary voltage was 4000 V. Polarity of the ionisation source was positive.

### 2.6 Respiration assay

The respiratory activity of the cells was measured using a 10-channel respirometer Micro-Oxymax^®^ (Columbus Instruments, United States). Tests were performed in 300-mL Micro-Oxymax glass vials under constant stirring (160 rpm, 28°C ± 2°C). The rate (μL/h) of О_2_ consumption was estimated. Automatic registration of respiratory activity parameters was conducted every 30 min for 14 days.

### 2.7 Microscopy

#### 2.7.1 Visualisation of lipid drops

Samples from the control and MLX-treated cultures were stained with Nile Red (Nanjing Dulai Biotechnology Co., Nanjing, China) and examined under an Axio Imager M2 microscope (Carl Zeiss Microscopy GmbH, Jena, Germany) according to the procedures and protocols described in (Mrunalini and Girisha, 2017).

#### 2.7.2 Surface topography and nanostructure of bacterial cells

The influence of MLX on the cell surface morphology and topography was investigated using an Asylum MFP-3D-BIO™ atomic force microscope (AFM, Asylum Research Inc., United States). AFM-scanning was in the tapping mode in air using an AC240TS silicon cantilever (50–90 kHz; 0.5–4.4 N/m). The root means square average roughness of the cell surface, the length and width, and the volume and surface area of the cells were calculated. The images were processed using the programme Igor Pro 6.22A. (WaveMetrics, United States).

#### 2.7.3 Transmission electron microscopy

Cells were harvested from the control and test cultures and fixed in 2.5% (w/v) in 0.1 M sodium cacodylate buffer (pH 7.2) for 2.5 h and post-fixed in 1% (w/v) osmium tetroxide in the same buffer. The fixed material was dehydrated through series of ethanol solutions to absolute ethanol saturated with uranyl acetate, and embedded in araldite. Thin sections were prepared on an ultratome (LKB, Sweden) and stained with lead citrate. Ultrathin sections were examined using a transmission electron microscope JEM-1400 (JEOL, Japan).

### 2.8 Energy dispersive X-ray spectroscopy

Suspensions of harvested cells in sterile water (without treatments with fixatives) were applied onto Formvar-coated and carbon-reinforced copper grids and air-dried. TEM with energy dispersive X-ray spectroscopy (EDX) with elemental mapping were performed using a JEM-1400 microscope (JEOL, Japan) equipped with energy dispersive X-ray analysis system (EDXA, Inca Energy-350, Oxford Instruments, United Kingdom), operating at accelerating voltage of 80 kV (tilt angle, 15°). The elemental maps were obtained by using AZtec software (Oxford Instruments, United Kingdom).

### 2.9 Zeta-potential of bacteria

The electrokinetic (zeta, or ζ-) potential was measured using a ZetaSizer Nano ZS analyzer (Malvern Instruments, United Kingdom) with Malvern ZetaSizer software, v. 2.2, and calculated based on the following equation:

$$\zeta =\frac{\eta u}{\varepsilon_{r}\varepsilon_{0}}$$

where ζ – zeta-potential, V; u – electrophoretic mobility, m^2^/Vs; η – viscosity, N/m^2^s; ε_0_ – dielectric permittivity in vacuum, F/m; ε_r_ – relative dielectric permittivity.

Before measurements, cells were washed twice with KNO_3_ buffer with pelleting of biomass using HERMLE Z200A (Hermle, Germany) centrifuge and re-suspended in the same buffer to OD_600_ 0.5 measured in a spectrophotometer Lambda EZ201 (Perkin-Elmer, United States).

### 2.10 Genomics

Whole-genome sequences of *G. alkanivorans* IEGM 1277 were obtained upon Next Generation Sequencing using a NovaSeq (Illumina, United States) sequencer.

### 2.11 *In silico* analysis of MLX decomposition products

The ecotoxicity of MLX and its bacterial degradation products was calculated using the ECOSAR (Ecological Structure Activity Relationships, https://www.epa.gov/) programme available in the software package EPI Suite TM (Estimation Programs Interface, EPA, United States). The ecotoxicity results were predicted based on the existing database on the toxic effects of organic compounds from various chemical classes.

### 2.12 Biological activity of MLX decomposition products

The biological activity profile of individual MLX metabolites was predicted using PASS (Prediction of Activity Spectra of Substances) online programme based on their structural formulas; the highest probability of bioactivity detection was taken as 1.0.

### 2.13 Biological potential of MLX decomposition products

The MICs of MLX degradation products against bacterial test cultures *Bacillus subtilis* АТСС 6633, *Micrococcus luteus* NCIMB 196, *Staphylococcus aureus* АТСС 25923, *Pseudomonas plecoglossicida* IEGM 2044 were assayed using the twofold serial dilutions method (Wiegand et al., 2008) in 96-well microplates incubated at 28°C (for *M*. *luteus* NCIMB 196, *P. plecoglossicida* IEGM 2044) or 37°C (*B. subtilis* АТСС 6633, *S. aureus* АТСС 25923) for 24 h. Cell viability was determined upon measuring the OD of formazan at 490 nm using a Multiskan Ascent plate spectrophotometer (Thermo Electron Corporaton, United States).

### 2.14 Statistical analysis

The validity of the experimental data was confirmed by control with the use of standard samples. The experiments were conducted in triplicate. Data analysis was conducted using Excel 2021 (Microsoft Inc., 2021), calculating the mean and standard deviation.

## 3 Results

### 3.1 Resistance of *Gordonia* strains to MLX

The MIC of the MLX against the representatives of *Gordonia* species under study varied in the concentration range from 0.625 to 5.000 and above g/L (Table 1). The most resistant to high (MIC ≥5.000 g/L) MLX concentrations were 5 strains belonging to *G*. *alkanivorans* IEGM 1277, IEGM 1398, *G*. *amarae* IEGM 720^T^, *G*. *rubripertincta* IEGM 101, and IEGM 113, predominantly isolated from oil-contaminated soils, using *n*-hexadecane, crude oil as the only carbon source (Catalogue of Strains, 2025). The high resistance of the identified strains to MLX exposure determines their potential use in the removal of this drug.

**TABLE 1:** MICs of the non-steroidal analgesic MLX against actinomycetes.

MLX concentration, g/L	Species	Strain
>5	*G*. *alkanivorans*	IEGM 1277, IEGM 1398
	*G*. *amarae*	IEGM 720^T^
	*G*. *rubripertincta*	IEGM 101, IEGM 113
5	*G*. *rubripertincta*	IEGM 733, IEGM 747, IEGM 749
2.5	*G*. *alkanivorans*	IEGM 1385
	*G*. *amicalis*	IEGM 1266
	*G*. *rubripertincta*	IEGM 102, IEGM 127, IEGM 1388
	*G*. *sputi*	IEGM 674^T^
1.25	*G*. *rubripertincta*	IEGM 96, IEGM 99, IEGM 106, IEGM 137, IEGM 1390
0.625	*G*. *rubripertincta*	IEGM 1392

### 3.2 MLX biodegradation by *Gordonia alkanivorans* IEGM 1277 cells and the predicted toxicity of the produced metabolites

**FIGURE 1:**
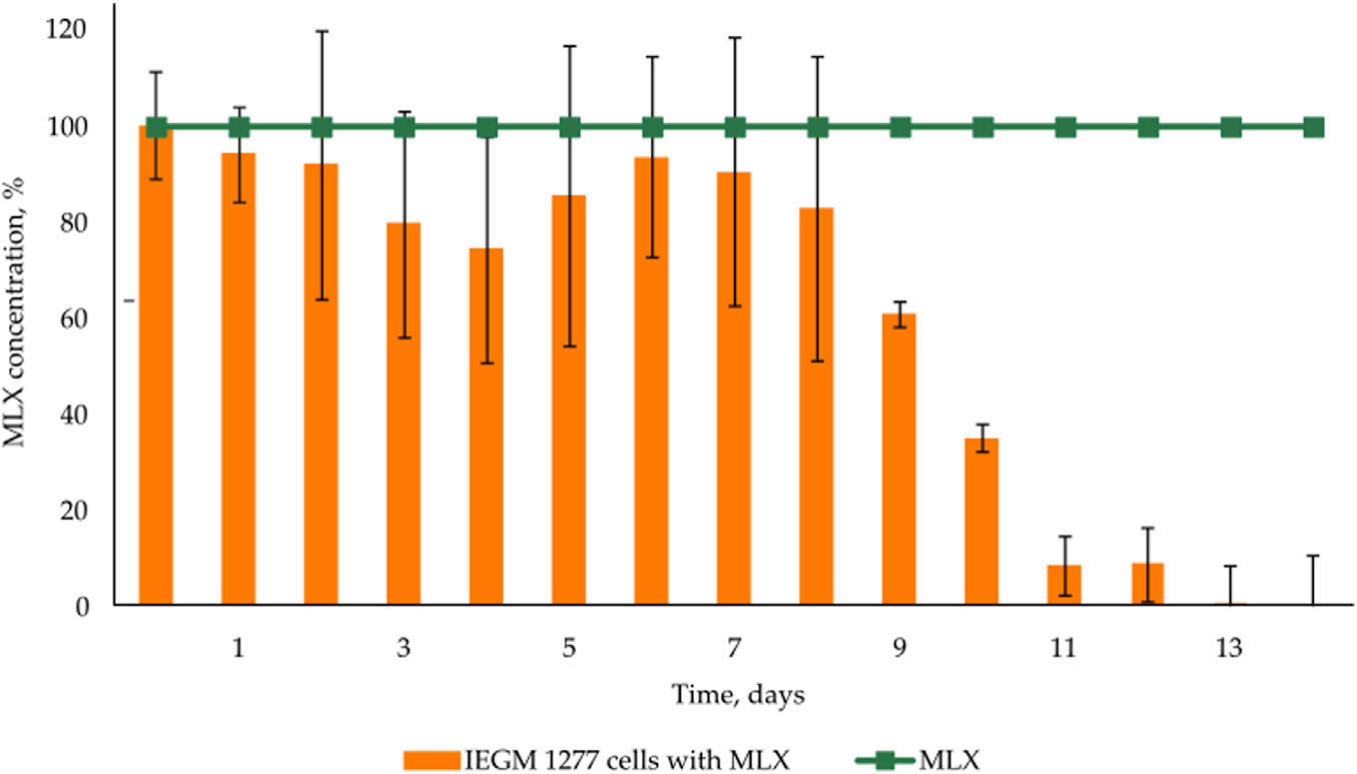
MLX depletion in the cultivation medium of *Gordonia alkanivorans* IEGM 1277.

**FIGURE 2:**
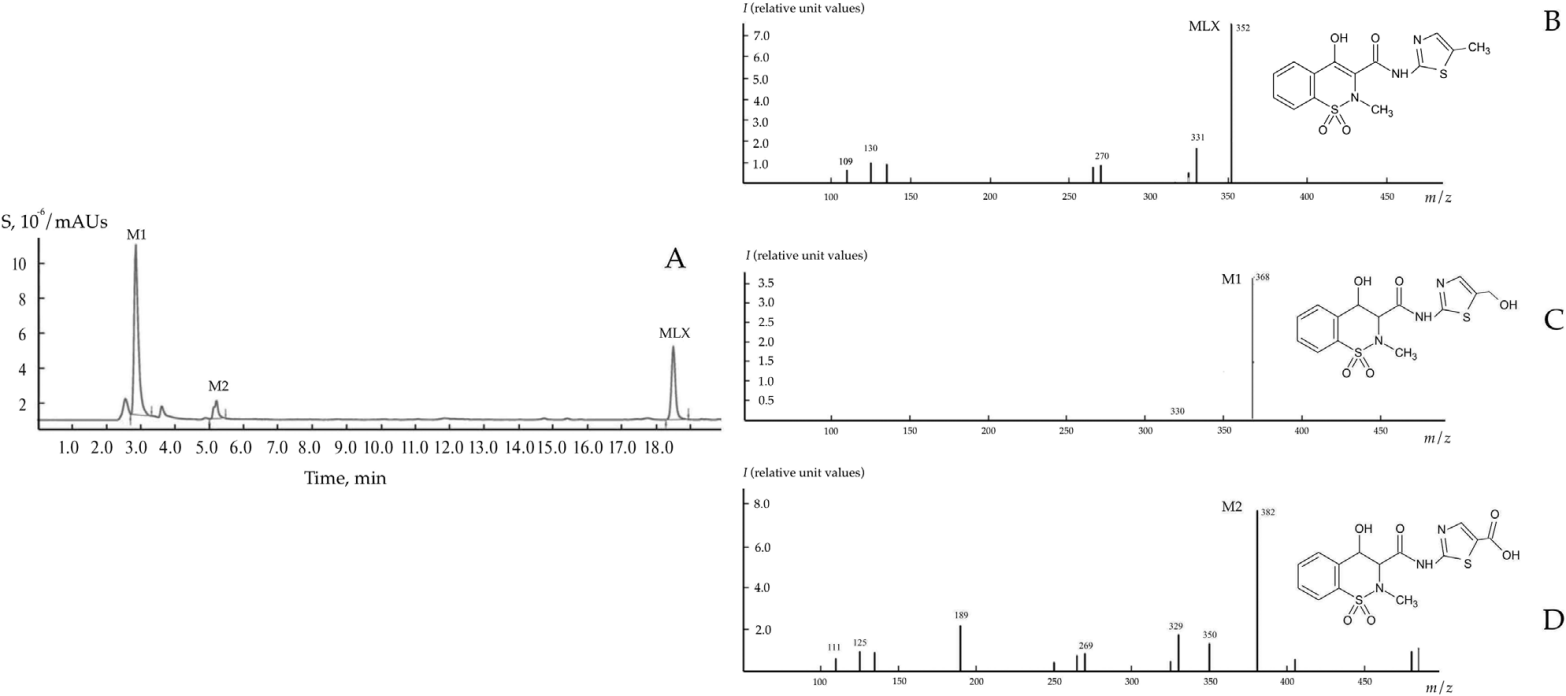
Chromatogram **(A)** and mass spectra **(B–D)** of MLX and its biodegradation products using *Gordonia alkanivorans* IEGM 1277. M1 – 5'-hydroxymethylmeloxicam; M2 – 5'-carboxymeloxicam.

**FIGURE 3:**
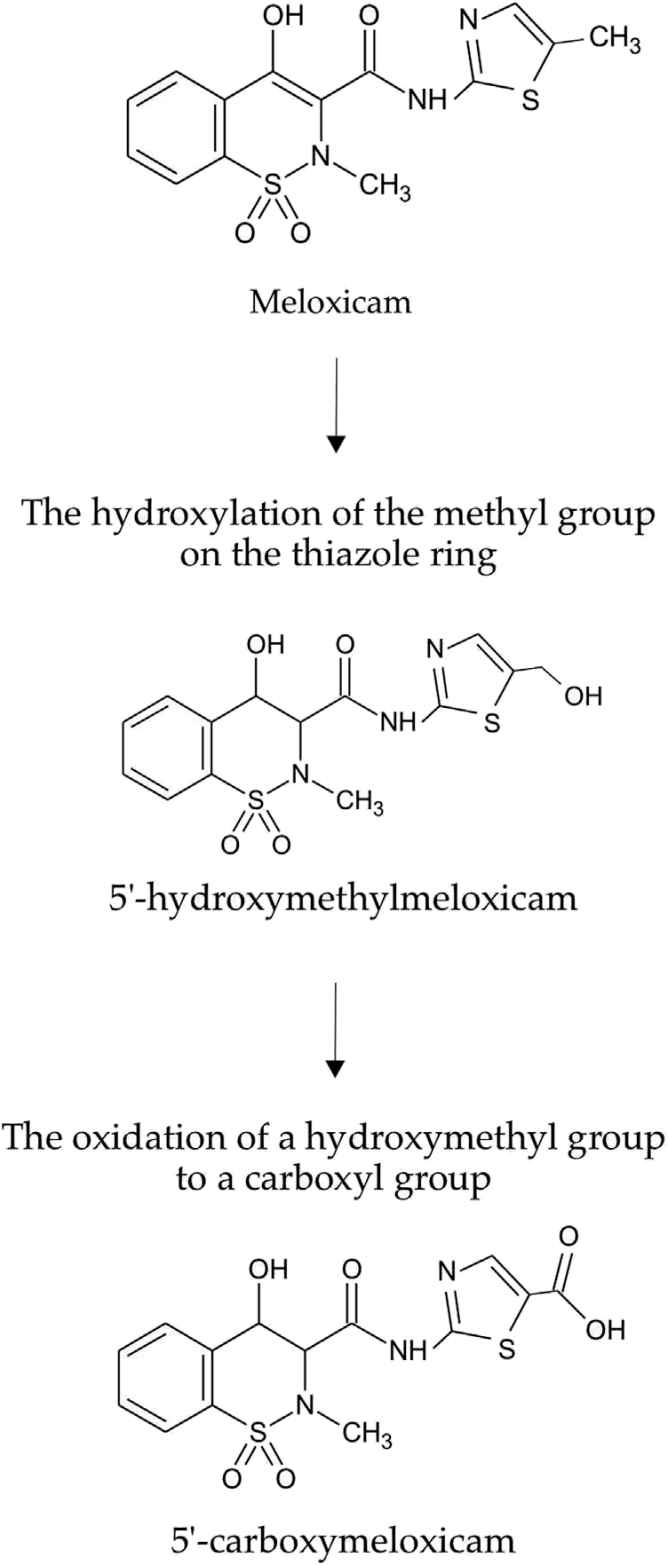
Metabolic decomposition of MLX using *Gordonia alkanivorans* IEGM 1277.

As was shown in preliminary experiments, significant biodegradation MLX was not the case for the concentrations from 20 to 50 mg/L. For further studies, we used the working MLX concentration of 10 mg/L to simulate environmentally relevant, near-peak loading conditions for studying biodegradation and cellular responses. Although this concentration exceeds typical wastewater levels, pharmaceutical pollutants can reach elevated concentrations near discharge sources (Gros et al., 2012; Jiménez et al., 2018; Wilkinson et al., 2024). Among the studied strains (Table 2), *G. alkanivorans* IEGM 1277 displayed the best degradative activity with respect to MLX judging from the complete exhaustion of MLX and served as the model object for subsequent experiments. The stable and rapid decline of the MLX level started after 7 days in cultures with *G. alkanivorans* IEGM 1277 with its half-disappearance by day 10 towards zero levels by the end of the experiments (Figure 1). Chromatographic analysis (Figure 2A) of the post-culture medium revealed compounds with *m*/*z* 368 (Figure 2C) and 382 (Figure 2D), corresponding to protonated metabolites of MLX: 5'-hydroxymethyl- and 5'-carboxymeloxicam. This indicates that MLX decomposition commences with a hydroxylation reaction, followed by oxidation of the side chain to a carboxylic acid (Figure 3).

**TABLE 2:** MLX content at 7 and 14 days.

Strain	MLX content, %	
	7 days	14 days
*G*. *alkanivorans* IEGM 1277	19.41	0.00
*G*. *alkanivorans* IEGM 1398	89.57	74.74
*G*. *rubripertincta* IEGM 733	64.17	70.46

According to *in silico* analysis, MLX is a compound with long-term concern for aquatic organisms, whereas the products of its bacterial conversion have less pronounced toxicity. As demonstrated by the predictive analysis, the toxicity indices of MLX metabolites were found to be 1.48–10.28 times less than those of MLX (Table 3). Our assays showed no significant antimicrobial activity of MLX metabolites against *B*. *subtilis* ATCC 6633, *M*. *luteus* NCIMB 196, *S*. *aureus* ATCC 25923, and *P*. *plecoglossicida* IEGM 2044: the MICs values exceeded 1.0 g/L. The prediction of pharmacological potential with the PASS online programme showed a low probability of detecting significant pharmacological effects. The probability score was 0.3 with a threshold reliability value of 0.7, indicating minimal biological activity of these compounds. MLX derivatives are probably not suitable for “green” chemistry due to the lack of antimicrobial activity.

**TABLE 3:** Predicted (eco)toxicity of MLX and its bacterial degradation products.

Compound	Fish		Daphnids		Green algae	
	ED_50_, 96 h	LD_50_, 96 h	ED_50_, 96 h	LD_50_, 48 h	ED_50_, 96 h	LD_50_, 96 h
MLX	0.49	494.14	25.38	832.64	8.10	12.73
5'-hydroxymethylmeloxicam	0.73	907.16	43.50	1,792.71	11.93	21.69
5'-carboxymeloxicam	5.02	4,930.46	255.65	8,131.59	83.26	128.33

LD_50_, median lethal dose for acute toxicity; ED_50_, median effect dose for chronic toxicity.

### 3.3 Response to MXL exposure

**FIGURE 4:**
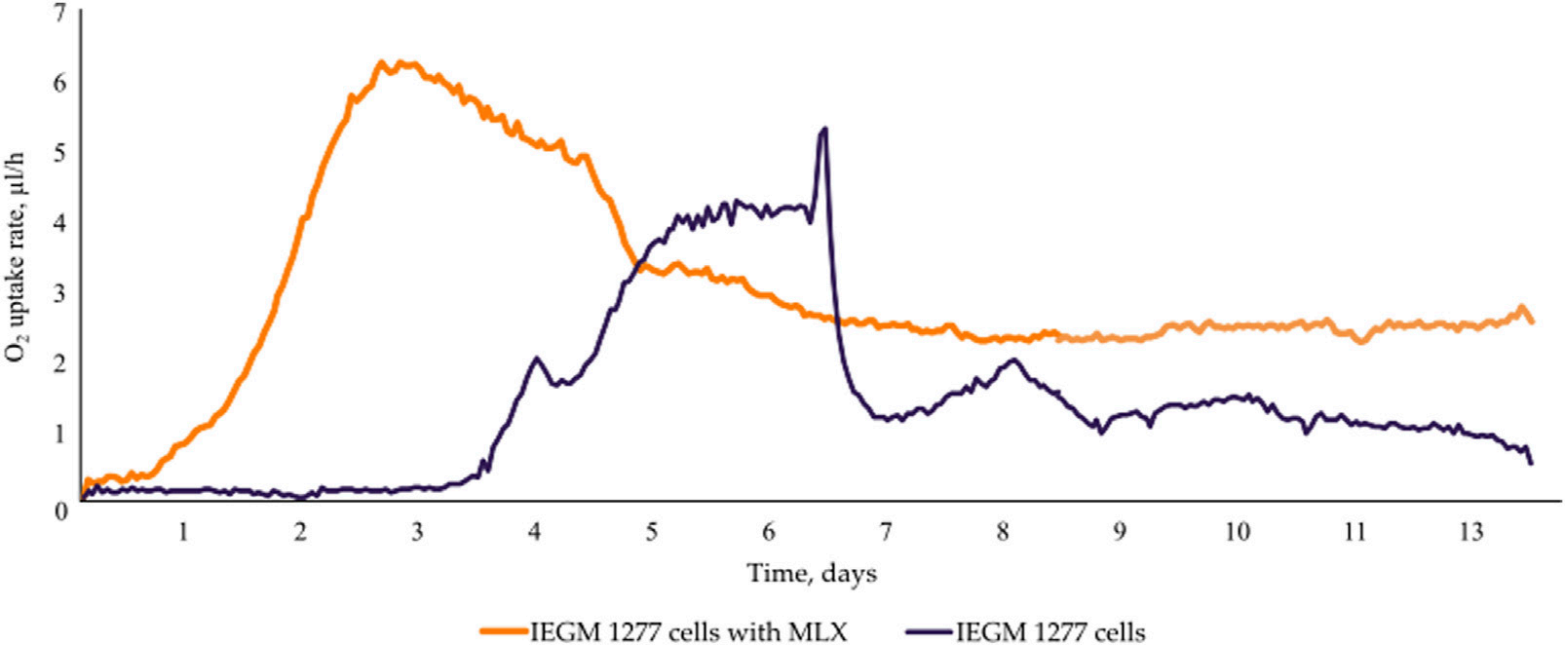
O_2_ consumption by *Gordonia alkanivorans* IEGM 1277 cells with/without MLX.

**FIGURE 5:**
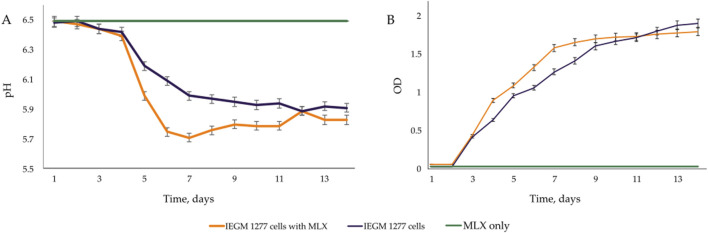
Changes in the **(A)** pH and **(B)** OD.

At the early stages of exposure to MLX, we observed a rapid activation of oxygen uptake by aerobic *G*. *alkanivorans* IEGM 1277 versus the control, MLX-free, cultures of this strain for which active respiration occurred after a 3-day lag. The observed increase in the maximum oxygen consumption by MLX-exposed cultures as compared to the control (Figure 4) could be reflect additional energy requirements to cope with oxidative stress caused by MLX. A faster acidification of the medium during MLX degradation than in MLX-free cultures (Figure 5A) could indicate the activation of processes to be involved in MLX decomposition and the accumulation of the MLX metabolites. As MLX was exhausted, the bacterial culture behaved similarly to the control, reaching the similar biomass yield (as judged from OD values) and stationary-phase onset (Figure 5B).

**FIGURE 6:**
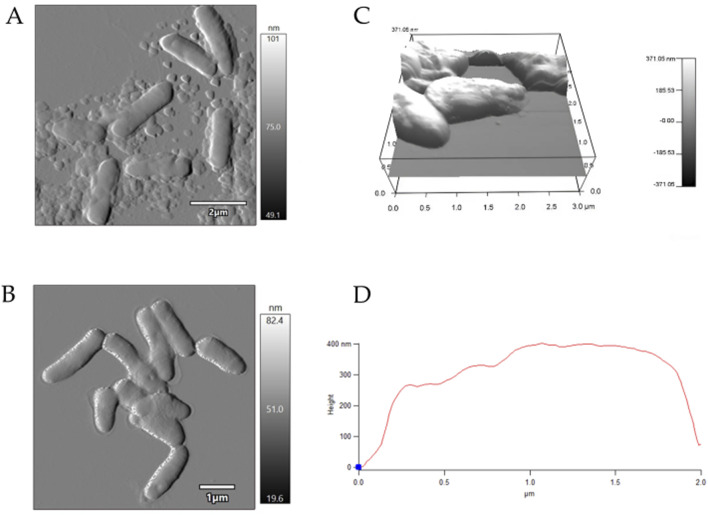
AFM-images of *Gordonia alkanivorans* IEGM 1277 cells with MLX: **(A)** at 7 days; **(B)** at 14 days; **(C)** 3D-image; **(D)** profile.

**FIGURE 7:**
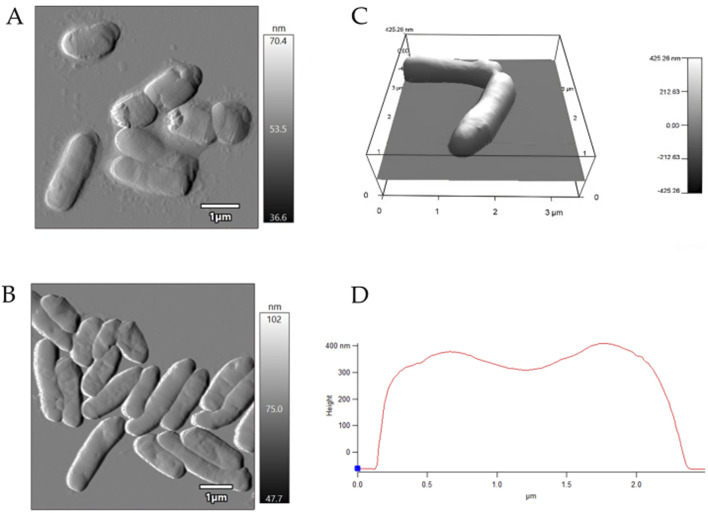
AFM-images of *Gordonia alkanivorans* IEGM 1277 cells without MLX: **(A)** at 7 days; **(B)** at 14 days; **(C)** 3D-image; **(D)** profile.

AFM examinations demonstrated that the exposure to MLX after 7 days was followed by a decrease in the surface area-to-volume (S/V) ratio of *G*. *alkanivorans* IEGM 1277 cells (Table 4) and its further increase by 14 days. The pattern is possibly due to the depletion of MLX from the medium, its more efficient degradation, and the release of less toxic metabolic products (5'-hydroxymethyl- and 5'-carboxymeloxicam). MLX caused insignificant elongation of bacterial cells: some cells had folds and protrusions (Figure 6C). On the contrary, *G*. *alkanivorans* IEGM 1277 cells in MLX-free cultures showed the relatively smooth and unaltered surface (Figures 7A,B).

**TABLE 4:** Morphometric changes in *Gordonia alkanivorans* IEGM 1277 bacterial cells under the influence of MLX.

Characteristic	7 days		14 days	
	With MLX	Without MLX	With MLX	Without MLX
Length, µm	1.83 ± 0.530	1.79 ± 0.280	1.65 ± 0.490	1.60 ± 0.350
Width, µm	0.61 ± 0.090	0.59 ± 0.030	0.83 ± 0.120	0.59 ± 0.040
Area (S), µm^2^	37.00 ± 4.400	53.50 ± 3.100	39.70 ± 3.500	14.50 ± 1.400
Volume (V), µm^3^	1.36 ± 0.011	1.23 ± 0.002	1.72 ± 0.015	1.21 ± 0.003
S/V, µm^−1^	27.07 ± 2.700	43.49 ± 2.120	22.69 ± 2.290	12.24 ± 1.160
Surface roughness, µm	0.12 ± 0.064	0.24 ± 0.067	0.11 ± 0.062	0.16 ± 0.087

**FIGURE 8:**
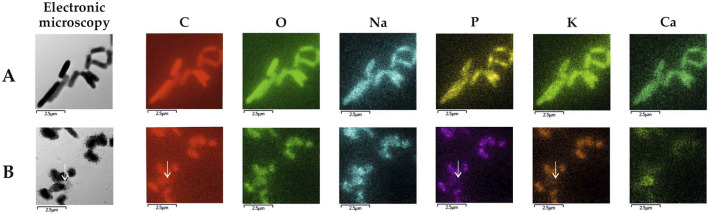
Elemental maps of *Gordonia alkanivorans* IEGM 1277 produced upon TEM-EDX microscopy: **(A)** without MLX; **(B)** with MLX. White arrows indicate a cell with depleted pools of some biogenic elements.

EDX-TEM microscopy examinations showed that the majority of cells in cultures with MLX maintained the pool of biogenic elements (carbon, oxygen, phosphorus) and potassium (Figure 8B) as in the control untreated culture (Figure 8A). Only a few cells in the MLX-exposed culture had the reduced level of these elements (Figure 8B, white arrow). It is noteworthy that the bacteria grown in the presence of MLX contained higher amounts of copper than in the control (Table 5), and an elevated copper level can be important for the activity of copper-containing monooxygenases and laccases, the enzymes catalysing reactions of oxidation of aromatic compounds (Solomon et al., 2014).

**TABLE 5:** The contents of chemical elements as derived from EDX-spectral mapping of whole fields with cells and cell-free space for *Gordonia alkanivorans* IEGM 1277 cultures with/without MLX.

Spectrum name	C	O	Na	Si	P	S	K	Ca	Cu
Without MLX	69.55	8.49	5.60	0.68	1.78	0.34	1.94	0.79	5.43
With MLX	81.78	6.02	0.43	0.65	0.48	0.12	0.80	0.24	9.30

**FIGURE 9:**
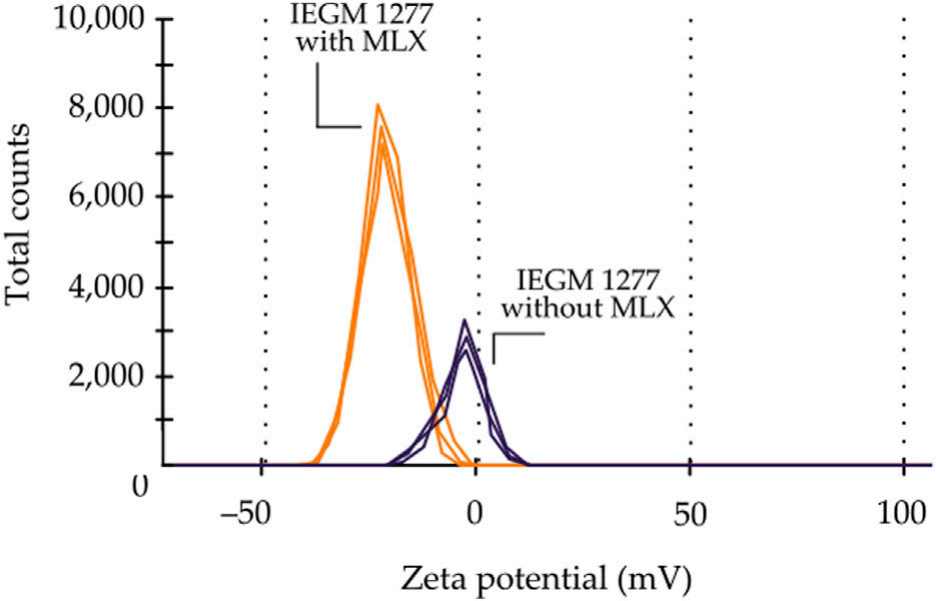
Changes in the ζ-potential of *Gordonia alkanivorans* IEGM 1277 with/without MLX.

The modification of the elemental composition of *Gordonia* was accompanied by a change in the biophysical parameter of bacterial cells known as the electrophoretic potential, which depends on the composition of the cytoplasmic membrane and the physiological state of the cells. Upon measuring the ζ-potential of control and MLX-exposed cells, a significant shift of the mean ζ-potential to the negative region by 15.12 mV, from −6.18 ± 0.200 mV to −21.300 ± 0.094 mV (Figure 9), was revealed. The increase in the absolute value of the electronegativity ζ-potential indicated the relative stability of a cellular system in the presence of MLX.

**FIGURE 10:**
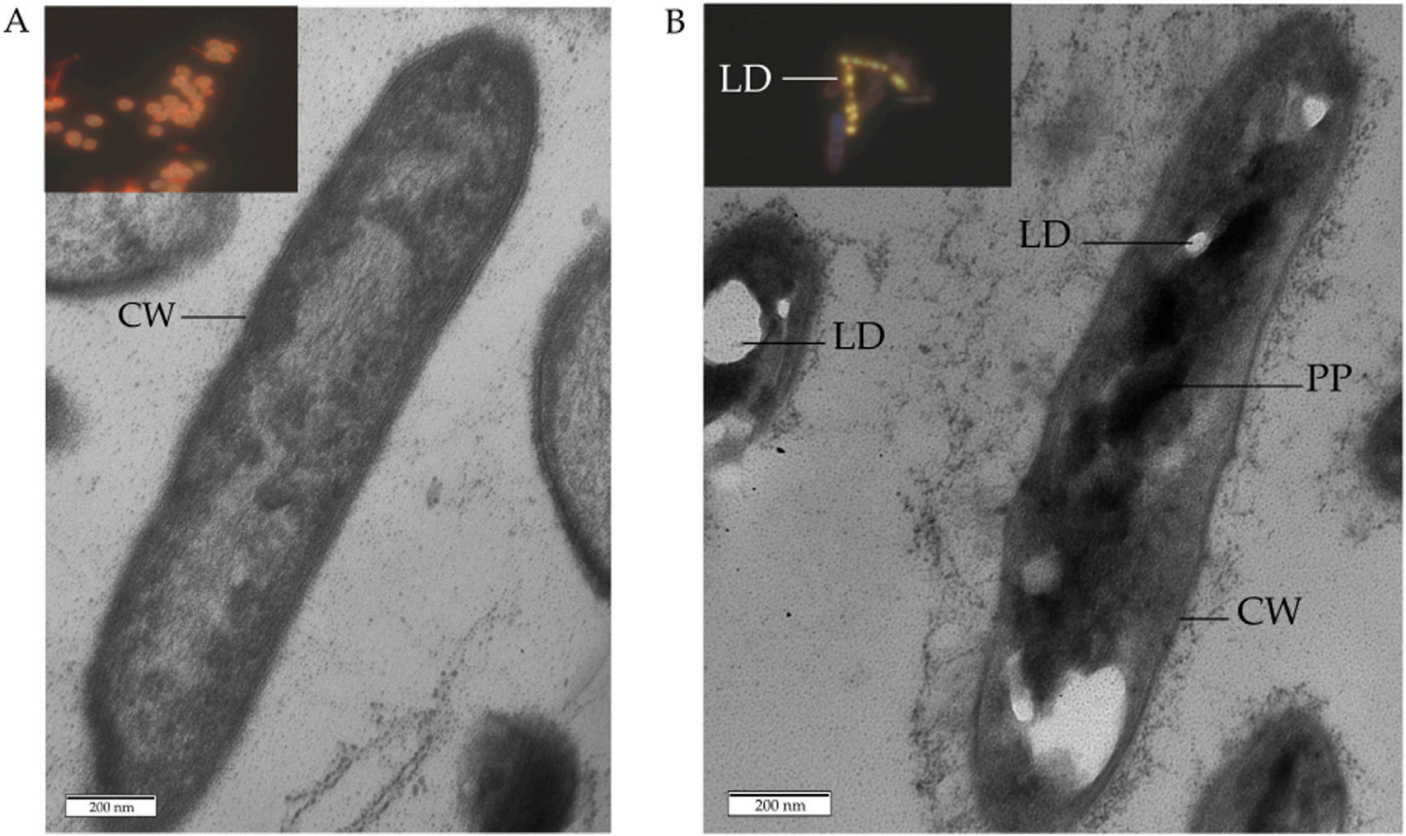
Transmission electron and fluorescent microscopy of *Gordonia alkanivorans* IEGM 1277 cells: **(A)** without MLX; **(B)** with MLX. Designations: LD–lipid droplets; PP–polyphosphate particles; CW–cell wall. Inserts to **(A,B)** shows images of cells after staining with Nile Red.

Comparative thin section TEM examinations proved the morphological integrity of cells in the control (Figure 10A) and MLX-exposed (Figure 10B) *G*. *alkanivorans* IEGM 1277 cultures as judged from the intact cell wall and intracytoplasmic structures. When grown in the presence of MLX, some cells showed the thick capsular layer with small granules around the cell wall and electron-dense particles to be composed of polyphosphate and electron-transparent droplets (Figure 10B) containing lipids, as supported upon staining with Nile Red (Figure 10B, insert). In the control cells, lipid droplets were absent, as well as electron-dense intracytoplasmic granules (Figure 10A).

Thus, the population was heterogeneous, with a substantial part of it tolerating the presence of MLX. The identified changes in *Gordonia* under the influence of MLX highlight the complexity of interactions with bacterial cells, as well as the need for further thorough investigation of the adaptive mechanisms developing in response to the action of toxic agents.

### 3.4 Full-genome sequencing

Full-genome sequencing of *G*. *alkanivorans* IEGM 1277 revealed features of the genome of the active MLX biodegrador (Table 6). The full-genome sequence is available in the DDBJ/ENA/GenBank databases under the numbers JASIRQ010000001-JASIRQ010000143. The genome size of IEGM 1277 was 5.1 Mb, the content of GC formed the majority (67.5%) of the genome in strain IEGM 1277. The percentage ratio of sequences with known and unknown function was 20:80, respectively. According to the obtained genomic data, chromosome IEGM 1277 contained 8 distinct regions (nucleotide sequences) responsible for encoding P450-dependent monooxygenases (Table 7). Table 8 presents the results of the genes and regulatory elements analysis associated with CYP450. Sequences encoding transcriptional regulators have been identified in the adjacent regions for 8 genes in this family, either in close proximity or immediately upstream of them. Cytochrome partner proteins, such as ferredoxin and ferredoxin-reductase, were found only in the vicinity of CYP450 genes No. 2, 3, 4, and 8 (Ugalde et al., 2018).

**TABLE 6:** Genome features of *Gordonia alkanivorans* IEGM 1277*.

Attribute	*G*. *alkanivorans* IEGM 1277
Genome size, bp	5,116,883
GC content, %	67.5
N50	148,056
L50	10
Number of contigs	143
Genes (total)	4,762
Genes (coding)	4,591
CDSs	4,707
RNA	55
tRNAs	48
ncRNAs	3
Pseudo genes	116
Genome coverage	672×

* According to the onlne service NCBI.

**TABLE 7:** Genes encoding CYP450 enzymes found in the *Gordonia alkanivorans* IEGM 1277 genome.

Gene no.	Contig ID	Gene location	Amplicon size, bp
1	NZ_JASIRQ010000002.1	323087–324397	1,310
2	NZ_JASIRQ010000004.1	94170–92782	1,388
3	NZ_JASIRQ010000005.1	227409–226072	1,337
4	NZ_JASIRQ010000008.1	163169–161919	1,250
5	NZ_JASIRQ010000013.1	115887–117302	1,415
6	NZ_JASIRQ010000015.1	29706–28456	1,250
7	NZ_JASIRQ010000020.1	32053–33330	1,277
8	NZ_JASIRQ010000049.1	1683–3,074	1,391

**TABLE 8:** Genetic surroundings of genes encoding CYP450 enzymes in *Gordonia alkanivorans* IEGM 1277.

Gene no.	Nearby genes	
	Upstream transcriptional regulators	Proteins
1	AcrR family	Enoyl-CoA hydratase; monooxygenase; oxygenase; NADH:flavin oxidoreductase/NADH oxidase; cell wall-binding protein
2	AcrR family	Ferredoxin reductases; ferredoxin, 2Fe-2S; glyoxalase/bleomycin resistance protein/dioxygenase
3	AcrR family	Ferredoxin reductase; oxidoreductase, dehydrogenase/reductases; O-succinylbenzoate synthase
4	GntR family	Ferredoxin reductases; ferredoxin, 2Fe-2S; dehydrogenases with different specificities; lipase
5	AraC family	Monooxygenase; lyase
6	LuxR family	Esterase; 3-hydroxybutyryl-CoA dehydrogenase; 3-hydroxyacyl-CoA dehydrogenase
7	AcrR family	Alcohol dehydrogenase; cyclase; aldehyde dehydrogenase
8	No	Ferredoxin reductase; ferredoxin, 2Fe-2S; Na^+^/H^+^ antiporter NhaA type

Using inhibitors of CYP450 enzyme activity, it has been confirmed that the oxidation process of MLX was catalysed by one of the enzyme complexes. Adding 1-aminobenzotriazole at concentrations ranging from 0.1 to 1 mM stopped the process of MLX biodegradation by IEGM 1277 cells, as evidenced by the presence of MLX in the post-culture medium after 14 days of exposure. This confirmed that the process of MLX biodegradation could be due to the activity of cytochrome P450-dependent oxygenases.

The findings extend the understanding of the molecular genetic basis of MLX biodegradation by *Gordonia* actinomycetes and set the stage for further analysis of gene expression levels to identify genes and enzymes that enhance the oxidation efficiency of MLX.

## 4 Discussion

Our study corroborates previous findings that underscore the substantial potential of microorganisms in the bioremediation of diverse pollutants, including pesticides, mycotoxins, biodegradable plastics, and heavy metals (Litvinenko, 2019; Taheur et al., 2019; Ramírez et al., 2023; Payanthoth et al., 2024). Understanding the biodegradation of specific toxicants necessitates detailed investigation into the underlying microbial and enzymatic mechanisms.

The isolation and characterisation of bacterial strains such as *B. subtilis* and *B. megaterium* (Xia et al., 2017; Shang et al., 2019), alongside the yeast *Cryptococcus podzolicus* (Wei et al., 2022), capable of degrading mycotoxins, provide valuable insights applicable to another challenge of pharmpollutant bioremediation. Similar to mycotoxins, many pharmaceutical compounds exhibit persistence and toxicity in the environment, necessitating efficient microbial degradation strategies. The elucidation of factors influencing mycotoxin biotransformation (including substrate concentration, temperature, pH, metal ions, pollutant source, and organic acids) via recombinant enzymes such as aldo-keto reductase from *Meyerozyma guilliermondii* (Zhang et al., 2024), alongside transcriptomic analyses revealing microbial response pathways (Wei et al., 2022), underscores the complexity and specificity of microbial detoxification mechanisms. These findings highlight the critical role of microbial communities and enzymatic systems in adapting to and transforming structurally diverse xenobiotics, including MLX. Understanding such parameters is essential for optimising bioremediation processes.

Moreover, the efficiency of degrading recalcitrant xenobiotics such as MLX varies significantly between ecosystems, influenced by substrate properties, environmental conditions, and microbial community composition (Prasad et al., 2009; Prasad et al., 2011). Consistent with observations in plastic biodegradation (Payanthoth et al., 2024), soil ecosystems typically harbour more diverse and metabolically versatile microbial assemblages compared to aquatic environments, enabling more rapid and complete degradation. This pattern extends to pharmaceuticals, where soil microbiota exhibit enhanced capacity for the mineralisation of anthropogenic micropollutants (Ivshina et al., 2015; Ivshina et al., 2019; Ivshina et al., 2021). Environmental factors including redox potential, nutrient availability, and temperature further modulate microbial activity and degradation kinetics, emphasising the necessity for ecosystem-specific bioremediation strategies. Integrating these ecological and biochemical insights is therefore essential for the development of effective bacterial-mediated biodegradation approaches targeting MLX and other pharmaceutical contaminants in diverse environmental matrices.

The main novel result of our study is the demonstrated ability of *Gordonia sensu stricto* to degrade MLX completely under the selected conditions. Our choice of the genus *Gordonia* for MLX biodegradation was informed by its phylogenetic proximity to the genus *Rhodococcus*, both classified within the order *Mycobacteriales*, class *Actinomycetes* (Goodfellow, 2014). Previous studies have demonstrated the capacity of *Rhodococcus* spp. to decompose drotaverine (Ivshina et al., 2015), diclofenac (Ivshina et al., 2019), and ibuprofen efficiently (Ivshina et al., 2021), supporting the hypothesis that *Gordonia* spp. Could exhibit similar metabolic capabilities toward NSAIDs. This hypothesis was substantiated in the present work, where *G. alkanivorans* IEGM 1277 effectively degraded MLX under the tested conditions.

Our study (Tables 6,7) has revealed the presence of distinct genes, responsible for encoding P450-dependent enzymes, the most important players in the process of biological oxidation of drugs, in the genome of *G. alkanivorans* IEGM 1277 (Guengerich, 2006). Based on these data (Table 8), we hypothesised that the genes encoding these CYP450s (No. 2, 3, 4, 8), ferredoxin, and ferredoxin-reductase formed a gene cluster that was potentially expressed as a polycistronic transcript and that these proteins are involved in a separate metabolic process, likely related to MLX oxidation. It is noteworthy that genes encoding CYP450 were also found in the genome of *R. rhodochrous* IEGM 757, the efficient biotransformation agent of oleanolic acid – a recalcitrant triterpenoid substance (Luchnikova et al., 2022). Other cytochromes in IEGM 1277 may be involved in complex biochemical and metabolic processes, allowing bacteria to survive and adapt in the absence of toxic MLX. These processes may involve the catabolism of lipids, fatty acids or amino acids, as well as the synthesis and maintenance of cell wall integrity, as evidenced by the presence of genes encoding the relevant enzymes. Other metabolic processes may be associated with the neutralisation of toxic organic compounds, as sequences coding for various transport proteins and other oxidoreductases have been found nearby the CYP450 genes. Transcriptomic analysis by Wei et al. (2022) demonstrated that *C. podzolicus* Y3 upregulates cytochrome P450 enzymes upon exposure to ochratoxin A, underscoring their central function in oxidative mycotoxin degradation. Concurrently, zinc finger proteins regulate genes associated with detoxification and stress responses, while heat shock protein 70 maintains cellular integrity under toxin-induced stress. In this regard, cytochromes play an important role since cytochromes catalyse the initial enzymatic breakdown, supported by regulatory and protective proteins. It is plausible that analogous molecular adaptation mechanisms are exhibited by *Gordonia* in response to pharmpollutants exposure. Although this study focused on a limited subset of actinomycete strains as potential MLX degraders, further systematic investigation is warranted to comprehensively assess the biodegradation capabilities across a broader microbial spectrum.

Properly selecting MLX-tolerant and degrading strains (Tables 1,2) from twenty *Gordonia* representatives was an important step: only a few strains could remove MLX, with *G*. *alkanivorans* IEGM 1277 being the most effective*.* Different strains within the same species, e.g., *G*. *alkanivorans,* displayed different rates of MLX decomposition (Table 2), which is consistent with the reported results of MLX biotransformation by various *B. subtilis* strains and representatives of the *Pseudomonas* genus (Prasad et al., 2009). Due to the limited number of reports on the microbial degradation of MLX, it is not possible to compare the capabilities of various microorganisms as its destructors. Complete disappearance of MLX (10 mg/L) was achieved over 14 days of cultivation in the RS mineral medium in our study. Using the selected *B. subtilis* and *P. putida* strains enabled the removal of 27%–41% of MLX from the initial 20 mg/L level over a shorter cultivation period in a nutritionally rich broth (Prasad et al., 2009). Furthermore, the efficiency of MLX transformation by the fungus *Cunninghamella blakesleeana* depended upon the medium composition: the maximum (ca. 80%) biotransformation occurred when the microorganism was cultivated in the medium containing glucose as the carbon source and ammonium nitrate as the nitrogen source (Prasad et al., 2011).

The RS medium was selected for this study due to its cost-effectiveness, widespread use in actinomycete research, and demonstrated efficacy in supporting the degradation of ibuprofen by related *Rhodococcus* species (Ivshina et al., 2021). Its composition, including readily available ammonium sulphate, dipotassium phosphate, and sufficient iron concentrations essential for the activity of cytochromes and oxidoreductases (Meenakshi et al., 2024; Wu et al., 2022). Furthermore, the use of a simple mineral medium, as opposed to complex organic alternatives, facilitates the isolation of valuable metabolites generated during the biodegradation of target compounds.

Concentrations of MLX in environmental samples have previously been reported in the limited number of papers, ranging from nanograms to micrograms per litre (Jiménez et al., 2018; Gros et al., 2012), as in the case of other pharmaceuticals (Boxall and Brooks, 2024). However, industrial processes can result in localised, transient spikes in the concentrations of pharmaceutical compounds which can be orders of magnitude higher than environmental levels (Pal, 2017). Therefore, the use of MLX in concentrations exceeding the environmental levels, as in this study and the previous studies (Prasad et al., 2009; Prasad et al., 2011), is justified in terms of worst-case environmental MLX contamination and of evaluations of the upper limit of bacterial tolerance and adaptation. The concentration of MLX in the medium should also be selected. Initial 20–50 mg/L levels in the medium were not suitable; the concentration of 10 mg/L turned out to be optimal for MLX biodegradation by *Gordonia* strains, as well as even for the 80% chemical degradation of MLX using the sonophotocatalytic system with ferrous tungstate (Xu et al., 2021). Therefore, the efficacy of the microbial utilisation of MLX depends on the nature of the microorganism used, the cultivation conditions, and the concentration of the target substance. The disappearance of MLX was correlated with its biodegradation into 5'-hydroxymethylmeloxicam and 5'-carboxymeloxicam (Figure 2), consistent with metabolites previously detected in (Prasad et al., 2009; Prasad et al., 2011), both of which exhibit predicted low toxicity (Table 3). It is noteworthy that microbial removal of toxic compounds does not invariably entail enzymatic detoxification; for instance, the elimination of ochratoxin A by *B. megaterium* has been attributed to cellular adsorption mechanisms rather than enzymatic degradation (Shang et al., 2019).

Overall, the results of our study, on the one hand, demonstrated the integrity of the majority of *G. alkanivorans* IEGM 1277 cells developing in the presence of MLX. This was evidenced by electron microscopy visualising intact cells and subcellular structures (Figure 10), as well as a stable negative ζ-potential (Figure 9), indicating the preservation of the mycolic acid layer in the cell wall (Wilson et al., 2001). In addition, TEM-EDX-based analysis revealed no change in nutrient pools in most MLX-adapted cells; a few cells lost carbon, phosphorus, sulphur, and potassium (Table 5; Figure 8). The depletion of these and other chemical element stores, which is a manifestation of the cytotoxic effect, has been demonstrated on mycobacteria subjected to harsh antibiotic therapy (Salina et al., 2024).

On the other hand, physiological assays (Figures 4, 5) and AFM studies (Figure 6) demonstrate the flexible response of *G. alkanivorans* IEGM 1277 to the presence of MLX, as well as the return of cells to baseline as MLX is depleted. The observed slight cell elongation (Table 4) may be due to impaired peptidoglycan synthesis, a similar morphological feature characteristic of cells exposed to some antibiotics (Punchenko et al., 2024). The extracellular substance visible in AFM-images (Figure 6) or thin sections (Figure 10) could contain biogenic surfactants or their mixture with *n*-hexadecane and MLX. Biosurfactant synthesis enhances substrate bioavailability and is considered a mechanism by which cells adapt to the effects of hydrophobic MLX (Yan et al., 2024). Among the specific morphological features of cells exposed to MLX is the appearance of intracytoplasmic droplets with lipid components (Figure 10). These droplets may serve as a ‘storehouse’ for carbon sources that are also utilised in the synthesis of mycolic acids in *Gordonia*. This process contributes to the protective function of the cell wall against toxicants (Marrakchi et al., 2014). In addition to acting as a carbon reserve, lipids may also be involved in protecting DNA from degradation (Zhang et al., 2017). Similar ultrastructural changes were observed in members of the genus *Rhodococcus* developing in the presence of different NSAIDs (Ivshina et al., 2019; Ivshina et al., 2021) or oleanolic acid (Luchnikova et al., 2022).

## 5 Conclusion

Screening of 20 actinomycetes strains of the *Gordonia* genus maintained in the Regional Specialised Collection of Alkanotrophic Microorganisms (www.iegmcol.ru) revealed 5 strains with high (MIC ≥5.000 g/L) tolerance to MLX. No direct correlation was found between the resistance of the strains to the ecotoxicant and their species affiliation.

The highest enzymatic activity against MLX was exhibited by *G*. *alkanivorans* IEGM 1277, which was capable of completely degrading MLX (10 mg/L) in the presence of *n*-hexadecane for 14 days. The presence of MLX in the cultivation medium of *Gordonia* induced metabolic, morphometric, and ultrastructural changes in *G*. *alkanivorans* IEGM 1277 cells, which could be considered as bacterial adaptive mechanisms and, consequently, an increase in their resistance to the toxic effects of the pharmaceutical pollutant. According to the chemical structure metabolites analysis of the strain IEGM 1277, the most common molecules were 5'-hydroxymethylmeloxicam and 5'-carboxymeloxicam, which were found to be substantially less toxic than MLX. Gene clusters for the synthesis of P450-dependent monooxygenases responsible for the initial oxidation of the MLX molecule were identified by genomic mining. The novel and significant finding of this study is that the representative of the genus *Gordonia* is capable of efficient biodegradation of MLX. The findings of this study offer valuable insights into the ecological function of this group of actinomycetes as subjects of ecological rehabilitation in the detoxification of natural ecosystems. The study establishes the foundation for the implementation of novel technical solutions for advanced wastewater treatment in pharmaceutical production and the disposal of dangerous pharmaceutical waste.

## Data Availability

The datasets presented in this study can be found in online repositories. The names of the repository/repositories and accession number(s) can be found below: https://www.ncbi.nlm.nih.gov/, JASIRQ010000001-JASIRQ010000143.
